# Molecular evolution and expression of opsin genes in Hydra vulgaris

**DOI:** 10.1186/s12864-019-6349-y

**Published:** 2019-12-17

**Authors:** Aide Macias-Muñoz, Rabi Murad, Ali Mortazavi

**Affiliations:** Department of Developmental and Cell Biology, University of California, Irvine, CA 92697 USA

**Keywords:** Cnidaria, Opsin, Gene expression, Phototransduction, Phylogenetics

## Abstract

**Background:**

The evolution of opsin genes is of great interest because it can provide insight into the evolution of light detection and vision. An interesting group in which to study opsins is Cnidaria because it is a basal phylum sister to Bilateria with much visual diversity within the phylum. *Hydra vulgaris* (*H. vulgaris*) is a cnidarian with a plethora of genomic resources to characterize the opsin gene family. This eyeless cnidarian has a behavioral reaction to light, but it remains unknown which of its many opsins functions in light detection. Here, we used phylogenetics and RNA-seq to investigate the molecular evolution of opsin genes and their expression in *H. vulgaris*. We explored where opsin genes are located relative to each other in an improved genome assembly and where they belong in a cnidarian opsin phylogenetic tree. In addition, we used RNA-seq data from different tissues of the *H. vulgaris* adult body and different time points during regeneration and budding stages to gain insight into their potential functions.

**Results:**

We identified 45 opsin genes in *H. vulgaris*, many of which were located near each other suggesting evolution by tandem duplications. Our phylogenetic tree of cnidarian opsin genes supported previous claims that they are evolving by lineage-specific duplications. We identified two *H. vulgaris* genes (*HvOpA1* and *HvOpB1*) that fall outside of the two commonly determined *Hydra* groups; these genes possibly have a function in nematocytes and mucous gland cells respectively. We also found opsin genes that have similar expression patterns to phototransduction genes in *H. vulgaris*. We propose a *H. vulgaris* phototransduction cascade that has components of both ciliary and rhabdomeric cascades.

**Conclusions:**

This extensive study provides an in-depth look at the molecular evolution and expression of *H. vulgaris* opsin genes. The expression data that we have quantified can be used as a springboard for additional studies looking into the specific function of opsin genes in this species. Our phylogeny and expression data are valuable to investigations of opsin gene evolution and cnidarian biology.

## Background

The evolution of opsin genes has been the subject of many studies because opsins play an essential role in vision and light detection. Much research has focused on deciphering the opsin phylogenetic tree in an effort to better understand the evolution of eyes and vision [1–4]. Visual opsin genes often encode G-protein coupled receptors that initiate the phototransduction cascade, a mechanism by which light information is converted into an electrical signal to be interpreted by the brain. Visual opsins bind a light-sensitive retinal chromophore (11-*cis*-retinal in vertebrates) that changes its conformation from 11-*cis* to all-*trans* when activated by light [5]. In addition to light detection, opsin proteins can partake in other roles supporting vision. For example, vertebrate retinal G protein-coupled receptor (RGR) and squid retinochrome function in chromophore transport and regeneration by photoisomerizing all-*trans* retinal to 11-*cis*-retinal [6–8]. Moreover, opsins have also been found to function in extraocular light detection and light-independent behavior such as temperature sensation and hearing [9]. Their conservation in animal species and roles in sensory perception make the opsins an interesting gene family to study.

**Fig. 1:**
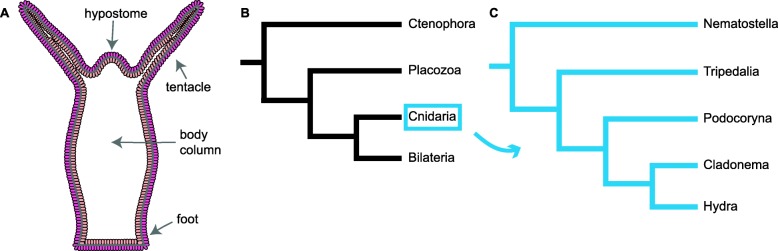
*H. vulgaris* body plan and cladograms. (**a**) Diagram depicting the *H. vulgaris* body plan which consists of the hypostome, tentacles, body column and foot. The *H. vulgaris* body is made up of two epithelial layers, the endoderm (light orange) and the ectoderm (bright pink). (**b**) Animal cladogram adopted from [13]. (**c**) Cnidaria cladogram inferred from [14, 15] to include only the species we used in this study, this is not a complete tree

A species in which to further investigate opsins is *Hydra* due to its basal location and role as a model organism. For over 270 years, *Hydra* has been used to address questions in multipotency, cell organization, neurogenesis, and regeneration [10]. The availability of a reference genome has facilitated studies of molecular evolution, gene expression, and gene functions [11, 12]. *Hydra* is a fresh-water polyp with a simple body plan made up of two epithelial layers, the endoderm and ectoderm (Fig. 1a). The *Hydra* body consists of a foot used to attach to substrate, body column, tentacles used to catch prey, and a hypostome (often referred to as the head). *Hydra* is capable of asexual reproduction by budding, during which a bud forms from the body column and develops in 10 stages until a small complete animal detaches from the parent [16]. Moreover, *Hydra* is of interest due to its ability to regenerate its head and foot when bisected [17–20]. *Hydra* can even regenerate from grafts and cell aggregates [21–23]. *Hydra* belongs to the basal animal phylum Cnidaria, which also includes jellyfish, sea anemones, and corals. Cnidaria is the sister group to Bilateria and also uses opsin-based phototransduction (Fig. 1b) [24, 25]. Until recently it was believed that Cnidaria was the most ancestral lineage capable of opsin based phototransduction [24, 26]. However, a recent study found that a ctenophore species possesses and expresses opsins with a conserved chromophore-binding site and found RNA-seq evidence for homologs of other components of the phototransduction cascade [27].

Even if Cnidaria is not the most ancestral group to use opsins, it is still a unique group to investigate opsin molecular evolution and gene expression due to high rates of lineage-specific duplications and the presence of eyes in the phylum. An early study of cnidarian opsins suggested that opsins had undergone several duplications in early hydrozoan evolution [28]. Investigation of opsins in a cubozoan genome found further evidence of rapid lineage- and species-specific duplications [29]. Further, Cnidaria are the most primitive invertebrates to possess eyes and, unlike bilaterian invertebrates that possess rhabdomeric photoreceptors, cnidarians have ciliary photoreceptors similar to vertebrates [30, 31]. Some cnidarians, such as box jellyfish of the class Cubozoa, even have complex camera-type eyes and use visual cues to navigate [32–34]. Recently, it was discovered that in Cnidaria alone, eyes have evolved independently a minimum of eight times and visual phototransduction has arisen through co-option of non-visual opsins [35]. While some cnidarian species have eyes and others do not, opsins are expressed extraocularly and eyeless cnidarians possess light-detecting abilities [28, 29, 36, 37]. As an example, corals and sea anemones use light cues for reproductive behaviors [38, 39]. These discoveries highlight the importance of further understanding the evolution and potential function of opsins in these gelatinous creatures.

*Hydra* is an example of a cnidarian species that has many opsins and lacks eyes but has a behavioral response to light. It has been suggested that opsin studies in *Hydra* may shed light on the evolution of visual pigments in more derived animals [36]. An early study of opsins in Cnidaria discovered 63 opsin genes in *H. magnipapillata* v. 1.0 [28]. Suga et al. and ﻿Liegertová et al. found that *Hydra* opsins cluster into 2 and 3 groups respectively [28, 29]. Note that the *Hydra* 2.0 Genome Project found that *H. magnipapillata* is the same species as *H. vulgaris*. While lacking eyes, *Hydra* undergo a shortening and lengthening response to light that depends on the light intensity and wavelength [40, 41]. Furthermore, opsins play an important role in *Hydra* feeding and defense because an opsin, *HmOps2*, is responsible for discharging the cnidocytes [25]. *HmOps2* co-localized with a ﻿cyclic nucleotide gated (CNG) ion channel gene (*HmCNG*) and an arrestin gene (*HmArr*) both necessary for the transmission and termination of the phototransduction cascade in ciliary photoreceptors [24]. Pharmacological inhibition of CNG diminished the behavioral response of *Hydra* to bright-light proving that CNG channels play a role in cnidarian phototransduction and suggest that opsins and CNG were present in the common ancestor of Cnidaria and Bilateria [25]. In addition, a previous study of *Hydra* transcriptomics found that genes upregulated in the hypostome, tentacles, and foot were enriched for functions in G-protein coupled receptors further suggesting that opsins, which belong to this group, may have crucial functions in *Hydra* [42].

While *Hydra* uses opsins, CNG, and arrestin, it remains to be explored which other components of the phototransduction cascade *Hydra* possesses. Cnidarian opsins are similar to vertebrate ciliary opsins so we expected to see ciliary phototransduction genes co-expressed with one or more opsin genes. Ciliary and rhabdomeric photoreceptors are similar in that the general transduction pathway is the same beginning with activation by rhodopsin, transduction via G-protein coupled receptor and ion channels, and finally termination. However, some of the messenger genes that they employ vary. In *Drosophila melanogaster* (a model for invertebrate phototransduction), activation of rhodopsin by light causes the release of G*α*q which activates ﻿phospholipase C (PLC) [43]. Light-detecting rhodopsin is comprised of an opsin protein bound to a retinal molecule known as a chromophore, 11-*cis*-3-hydroxyretinal in *D. melanogaster* and 11-cis-retinal in mammals [5]. The chromophore is transported to the photoreceptor cell by a retinal binding protein, ﻿cellular retinaldehyde-binding protein (CRALBP) in mammals and ﻿prolonged depolarization afterpotential is not apparent (PINTA) in *D. melanogaster* [44, 45]. The transduction in *D. melanogaster* is carried out by Ca^2+^-permeable transient receptor potential (TRP) channels that cause depolarization of the cell [46, 47]. Finally, phototransduction is terminated when the activated rhodopsin (metarhodopsin) binds arrestin or is phosphorylated by rhodopsin kinase [48–50]. In vertebrates, activated rhodopsin works through GTP-binding transducin which releases Gt*α* and binds guanosine monophosphate phosphodiesterase (GMP-PDE) [51]. Instead of TRP, opening of cyclic nucleotide gated ion channels (CNG) cause the photoreceptor cell to hyperpolarize [51]. Similar to ciliary cells, rhodopsin kinase and arrestin terminate the cascade by deactivating rhodopsin [51]. In addition, in vertebrates, G Protein-coupled receptor kinase 1 (GRK1) and regulator of G protein signaling 9 (RGS9) regulate G protein signaling while recovering inhibits phosphorylation of light-activated rhodopsin [51].

In this study, we use an improved *Hydra* reference genome (*Hydra* 2.0 Genome Project) with augmented gene models and an ab initio transcriptome to investigate the molecular evolution of opsin genes in *H. vulgaris*. As previous studies have identified opsin genes in *Hydra* and generated cnidarian opsin phylogenies, we hypothesized that we might detect a similar number of previously identified genes and detect lineage-specific duplications with *H. vulgaris* opsins forming two groups [28]. However, since we are working with an updated genome and improved gene models, we also expected to find some variations from previous studies. We identified 45 opsins in *H. vulgaris* and found that many opsin genes are located in tandem. Our phylogeny provides support for lineage-specific opsin duplications in Cnidaria. We also found that two *H. vulgaris* opsins (*HvOpA1* and *HvOpB1*) do not group together in the phylogeny with other opsins. Next, we sought to explore the expression of opsin genes in the *H. vulgaris* body map and during regeneration and budding. We hypothesized that some opsins would have differential expression between tissues and that the opsins with high expression in adult hypostome and tentacle would undergo an increase during regeneration and budding. We expected highly expressed genes to increase during budding and regeneration because, if they function in the adult hypostome and tentacle, presumably their expression increases as these tissues develop. Our hypothesis was true for a subset of opsin genes. We were indeed able to identify genes that are upregulated in the *H. vulgaris* hypostome and tentacle and that increase in expression during budding and regeneration. Moreover, we determined that *HvOpA1* is the most highly expressed opsin and is expressed in all samples that we looked at, while *HvOpB1* is highly expressed in the hypostome and its expression increases during budding and regeneration. By exploring stem cell trajectories, [52] we found that *HvOpA1* and *HvOpB1* may have functions in nematoblasts and mucous gland cells respectively. Furthermore, by incorporating expression patterns of phototransduction genes, we identified opsins that are co-expressed with other phototransduction genes and imply these opsins may function in the *H. vulgaris* phototransduction cascade. We propose a model for phototransduction in *H. vulgaris* that has ciliary and rhabdomeric components based on expression patterns of phototransduction genes.

## Results

### Cnidarian opsins are evolving by linage-specific duplications

In order to investigate patterns of molecular evolution of opsins in *H. vulgaris,* we first curated opsin sequences in the recently released and improved genome, *Hydra* 2.0 Genome Project (formerly *H. magnipapillata*) [11]*.* By searching an ab initio transcriptome, phylogenetically-informed annotation (PIA) database [53], and an improved reference genome, we identified 45 opsin genes in *H. vulgaris* (Additional file 4: Table S1-S2). Our hypothesis that we would find a similar number of genes from previous studies was incorrect. Our result differed from that of 63 opsin genes found by Suga et al. [28] using the first genome release. Given the highly fragmented nature of the original assembly, we believe that the difference in opsin gene number between our studies is due to misalignments or haplotypes in the original assembly.

**Fig. 2:**
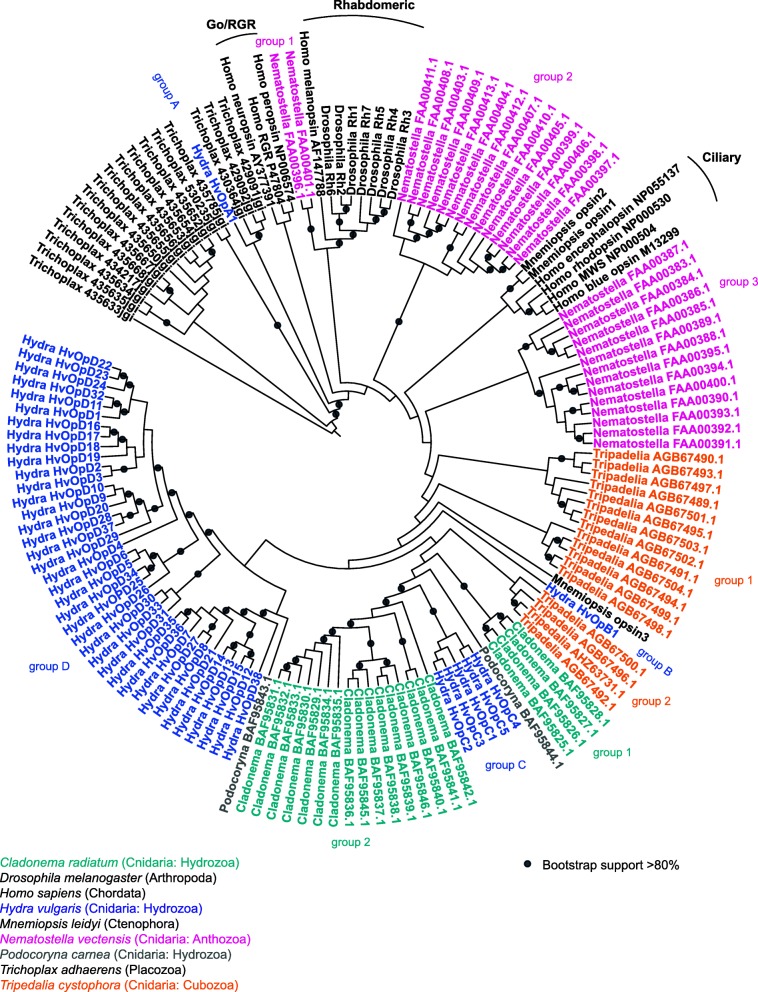
Cnidarian opsin phylogeny. Opsin phylogenetic tree generated using amino acid sequences for *Hydra vulgaris*, *Podocoryna carnea*, *Cladonema radiatum*, *Tripedelia cystophora*, *Nematostella vectensis, Mnemiopsis leidyi, Trichoplax adhaerens*, *Drosophila melanogaster* and *Homo sapiens*. Maximum-likelihood tree was generated using a LG + G + F model and 100 boostrap support

Next, we generated a cnidarian opsin phylogeny and included outgroups placozoa, humans, and *Drosophila* (Fig. 2). We made placozoa the root of the tree as determined by Feuda et al. [3, 54]. Based on previous studies, we expected to see lineage-specific duplications of opsins in Cnidaria with *Hydra* opsins forming two groups [28, 29] or we expected to see the opsin tree recapitulate the evolutionary history of the species (Fig. 1b-c). Our phylogenetic tree supported claims that opsins are evolving by lineage-specific duplications as *Hydra*, *Cladonema*, *Tripedalia*, and *Nematostella* opsins group together by species rather than opsin type (Fig. 2). Generally, the opsin phylogeny reflects the cnidarian cladogram with *Hydra*, *Cladonema* and *Podocoryna* closer together, next *Tripedalia*, and *Nematostella* a little further away (Fig. 2). Our opsin phylogeny provides support for previous suggested cnidarian opsin phylogenetic relationships. Similar to previous studies, we found ctenophore opsins *Mnemiopsis opsin1* and *opsin2* grouping together while *Mnemiopsis opsin3* branches separately (Fig. 2) [27, 54]. We also found that *Podocoryna* opsins do not group together [28] and that both *Cladonema* and *Tripedalia* opsins form 2 groups [28, 29].

We discovered some differences from previous studies as to the placing of a *N. vectensis* opsin group and two *H. vulgaris* opsins. Suga et al. and ﻿Liegertová et al. found that *N. vectensis* opsins cluster into 3 and 4 groups respectively [28, 29]. Here, we found that *Nematostella* opsins formed three groups; group 3 clusters with the cnidopsins, group 2 is outside of ciliary opsins (C-opsin) and cnidopsins, and group 1 is sister to rhabdomeric opsins (Fig. 2). We found that *H. vulgaris* opsins clustered into 2 main groups, but we also uncovered that 2 genes fall outside of these two large groups, so we refer to each of these its own group. *HvOpB1* (group B *Hydra* opsin) falls within *Mnemiopsis opsin3* and outside of a group of cnidopsins and *HvOpA1* (group A) is sister to a group of Placozoan opsins (Fig. 2). We refer to the other two groups as group C and group D. The overall mean distance between sequences in group C was 0.615, group D was 2.449 and between sequences from C and D together was 2.804. These results suggest that there is more variation between sequences in group D than group C.

As a majority of the cnidarian opsin genes form clusters, this suggests that opsin genes are expanding by linage-specific duplications rather than a large expansion in their common ancestor. In addition, we named our opsin genes based on location on the genome and found that many *H. vulgaris* opsin genes that are in close proximity in the genome are also next to or very close to each other on the phylogeny. As an example, opsin genes in group C (*HvOpC1–5)* are all on the same scaffold (Table S1) and next to each other on the phylogeny (Fig. 2). *HvOpD1–4* are also on the same scaffold but only *HvOpD2–3* group together. *HvOpD5–6* are on the same scaffold and branch together on the phylogeny. Other examples include *HvOpD9–10*, *HvOpD12–15*, *HvOpD16–19*, and *HvOpD22–24*. These groupings of genes on same scaffolds in the opsin phylogenetic tree suggest that *H. vulgaris* opsins could be expanding by tandem duplications (Fig. 2).

### Expression patterns of *H. vulgaris* opsins in the *Hydra* body, during budding, and during regeneration

**Fig. 3:**
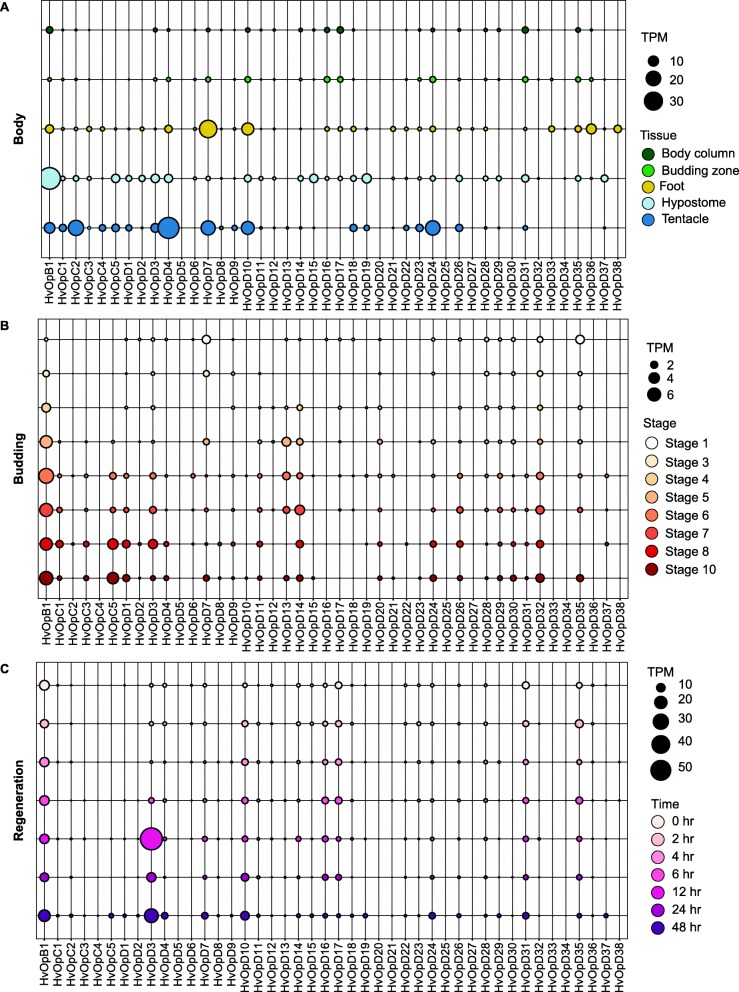
Opsin expression in the *H. vulgaris* body map, during budding, and during regeneration. (**a**) RNA-seq expression of opsins in *H. vulgaris* body column, budding zone, foot, hypostome, and tentacles measured in transcripts per million (TPM). (**b**) RNA-seq expression during *H. vulgaris* budding (asexual reproduction) at stages 1, 3, 4, 6, 7, 8, and 10 measured in transcripts per million (TPM). (**c**) RNA-seq expression during *H. vulgaris* head regeneration at times 0 h, 2 h, 4 h, 6 h, 12 h, 24 h, and 48 h measured in transcripts per million (TPM).

**Fig. 4:**
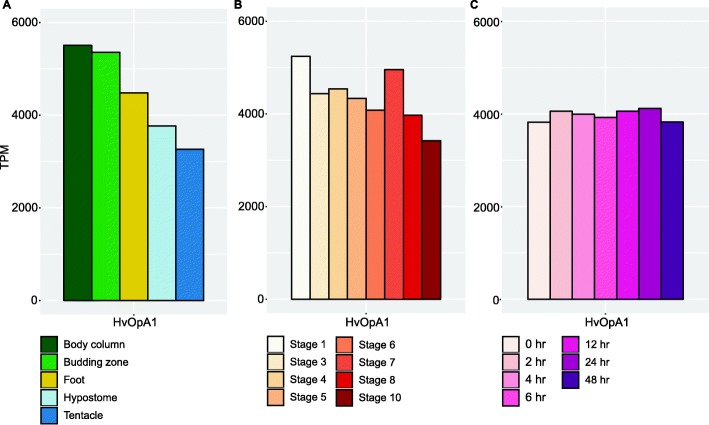
*HvOpA1* expression in the *H. vulgaris* body map, during budding, and during regeneration. (**a**) RNA-seq expression of opsin gene *HvOpA1* in *H. vulgaris* body column, budding zone, foot, hypostome, and tentacles measured in transcripts per million (TPM). (**b**) RNA-seq expression of opsin gene *HvOpA1* during *H. vulgaris* budding (asexual reproduction) at stages 1, 3, 4, 6, 7, 8, and 10 measured in transcripts per million (TPM). (**c**) RNA-seq expression of opsin gene *HvOpA1* during *H. vulgaris* head regeneration at stages 0 h, 2 h, 4 h, 6 h, 12 h, 24 h, and 48 h measured in transcripts per million (TPM)

Investigating the expression patterns of genes, especially when comparing tissues, can give some insight into their potential functions. We quantified the expression of the *H. vulgaris* opsins in the *H. vulgaris* body, during budding, and during regeneration [42]. Opsin genes that were expressed more highly (> 2 fold change) in the foot compared to other tissues were *HvOpD21*, *HvOpD27*, *HvOpD33*, *HvOpD36*, and *HvOpD38* (Fig. 3a; Additional file 1: Figure S1A). All of these genes are near each other on the opsin phylogeny and belong to an opsin gene cluster for which a *Podocoryna* opsin is an outgroup (Fig. 2). In the hypostome, the genes that were more highly expressed (> 2 fold change) relative to other tissues were *HvOpB1*, *HvOpD2*, *HvOpD11*, *HvOpD12, HvOpD14*, *HvOpD15*, *HvOpD19, HvOpD29*, *HvOpD32*, and *HvOpD37* (Fig. 3a; Additional file 1: Fig. S1A). These genes are not all near each other on the phylogeny, however *HvOp12, HvOp14* and *HvOp15* belong to a branch that includes genes located on the same scaffold and they have similar expression patterns across tissues (Fig. 4a). In the tentacle, opsin genes *HvOpC1*, *HvOpC2*, *HvOpC4*, *HvOpD4*, *HvOpD8*, *HvOpD9, HvOpD13*, *HvOpD22*, *HvOpD23*, and *HvOpD24* were expressed more highly (2x) relative to other tissues (Fig. 3a; Fig. 4a). *HvOpC1*–2 and *HvOpC4*, and *HvOp22–24* are next to each other in the genome, have similar sequences based on the opsin phylogeny, and have similar expression patterns across tissues. This suggests that these genes may have shared functions (Fig. 2, Additional file 1: Figure S1A).

We hypothesized that some of the genes that were expressed more highly in the hypostome and tentacles relative to other tissues would have expression that increased during budding and regeneration. For the hypostome, *HvOpB1* increases in expression during both budding and regeneration (Additional file 1: Figure S1A-C). *HvOpD2* and *HvOpD37* increase in expression during regeneration but do not show a temporal trend during budding (Fig. S1B-C). Conversely, *HvOpD14* and *HvOpD32* increase in expression during budding but do not have a directional change during regeneration (Additional file 1: Figure S1B-C). For the tentacle, *HvOpD4* increases during both regeneration and budding. *HvOpD13* only increases during budding while *HvOpD24* and *HvOpC2* increase during regeneration. These findings are interesting because *HvOpB1* is one of the most highly expressed genes in the hypostome and *HvOpC2, HvOpD4*, and *HvOpD24* are some of the most highly expressed genes in the tentacle and these four genes all show trend of increasing either in budding, regeneration, or both. High expression of a gene in a body part implies that the gene has a particular function specific to that tissue. These genes likely play an important function in the *Hydra* head. Only a subset of opsin genes increase in expression in budding and regeneration. Some genes may turn on later in the adult. It is important to note that *HvOpB1* falls outside of the two *H. vulgaris* opsin gene groups C and D. Instead, *HvOpB1* serves as an outgroup to all Hydrazoan opsins and one group of the *Tripedalia* opsins.

While *HvOpB1*, *HvOpC2*, *HvOpD4*, and *HvOpD24* are expressed highly in the *H. vulgaris* head region and have dynamic expression during budding and regeneration, we found another candidate gene for further potential function investigation due to its very high expression in *H. vulgaris*. *HvOpA1* is expressed almost 200-fold more than the other opsin genes (Fig. 4). We did not detect a significant difference in expression between body parts nor during different stages and times of budding and regeneration. The high expression of this gene throughout the *H. vulgaris* body suggests that it is a gene of importance with a general function. Similar to *HvOpB1*, *HvOpA1* does not fall within the *H. vulgaris* opsin gene clusters. Instead, *HvOpA1* groups with Placozoan opsins (Fig. 2).

**Fig. 5:**
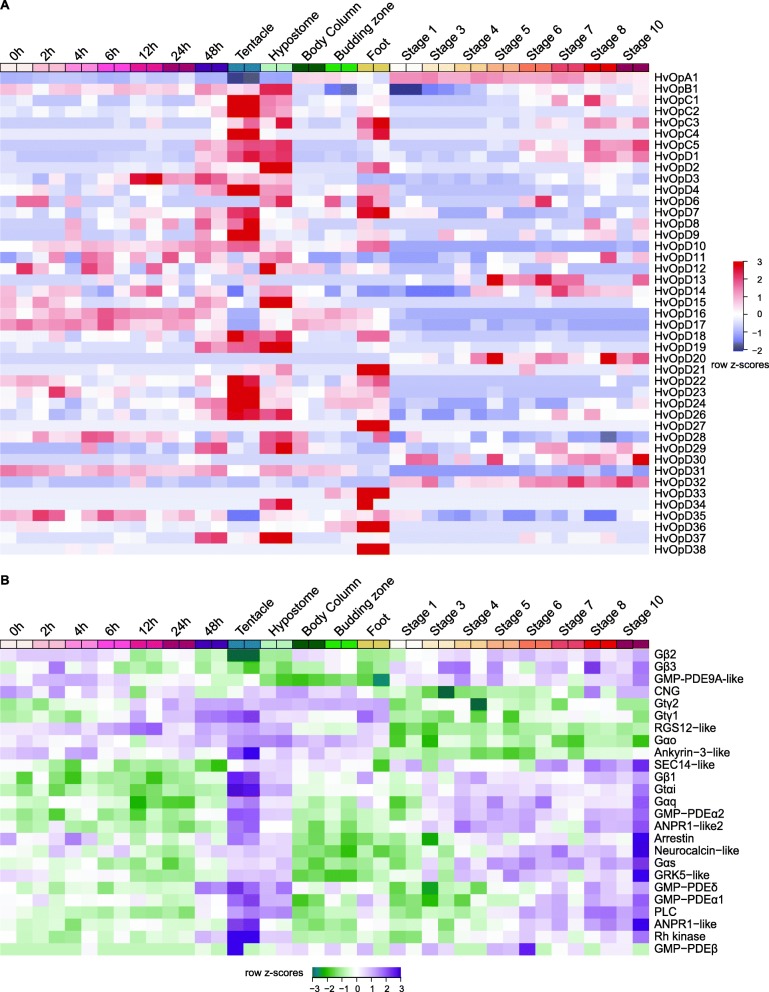
Opsin and phototransduction gene expression across all samples. (**a**) Heatmap showing RNA-seq row z-scores for opsin genes across all samples used in this study. 0 h–48 h represent samples of head tissue during different time points in regeneration; tentacle, hypostome, body column and foot are adult tissues; stage 1–10 are stages of budding during asexual reproduction. *HvOpD5* and *HvOpD25* are missing because they are not expressed in any of the samples. (**b**) Heatmap showing RNA-seq row z-scores for phototransduction genes across all samples used in this study

To increase our power, we also looked at opsin expression across all samples used together (Fig. 5a; Additional file 2: Figure. S2). From this analysis we notice three sets of genes that are upregulated in the hypostome, tentacle or foot. According to gene expression z-scores across all samples *HvOpB1*, *HvOpD3*, *HvOpD11*, *HvOpD15*, *HvOpD19*, *HvOpD29*, and *HvOpD37* have higher expression in the hypostome compared to other tissue types and also increased during budding. *HvOpC1*, *HvOpC2*, *HvOpC3*, *HvOpC4*, *HvOpC5*, *HvOpD1*, *HvOpD4*, *HvOpD7*, *HvOpD8*, *HvOpD9*, *HvOpD10*, *HvOpD16*, *HvOpD18*, *HvOpD22*, *HvOpD23*, *HvOpD24*, and *HvOpD26* group together as having similar expression patterns and are more highly expressed in the tentacles compared to other tissue types and time points in budding and regeneration (Fig. 5a; Additional file 2: Fig. S2). *HvOpD21*, *HvOpD27*, *HvOpD33*, *HvOpD36*, and *HvOpD38* are more highly expressed in the foot compared to other tissue types and time points in budding and regeneration (Fig. 5a; Additional file 2: Figure S2). For the most part, an analysis comparing across all samples had similar patterns of gene expression as pairwise comparisons between tissue types.

### Phototransduction cascade genes in *H. vulgaris*

In order to detect whether any of these opsins might function similar to vertebrate ciliary or invertebrate rhabdomeric opsins, we searched the *Hydra* genome for phototransduction genes using ﻿*M. leidyi* sequences following the example of ﻿Schnitzler et al. [27]. As mentioned above, cnidarians are of interest because they are basal invertebrates with ciliary opsins similar to vertebrates, thus we expected to find evidence of ciliary phototransduction cascade components.

In vertebrates and *Drosophila*, the chromophore binds opsins at a conserved retinal-binding lysine in the seventh transmembrane helix. In order to identify which of the *H. vulgaris* opsins may function in phototransduction, we investigated which had the conserved lysine necessary for chromophore binding. We found that all opsins except five have the lysine amino acid necessary for phototransduction. The five opsins missing the lysine were: *HvOpA1*, *HvOpB1*, *HvOpD4*, *HvOpD9*, and *HvOpD26* (Additional file 4: Table S1).

In terms of ciliary components, *H. vulgaris* differed from *M. leidyi* in that the top hit to G-alpha-i subunit is a G-alpha-o subunit (*Gαo*) (Table 1). Although we did not have an exact predicted protein match, Gαo and Gαi belong to the same Gα protein subfamily and are expected to have similar functions in signal transduction [55]. *H. vulgaris* also had two genes similar to Transducin G-gamma-t1 which we refer to as *Gtγ1* and *Gtγ2*, two GMP-PDE alpha rod genes (*GMP-PDEα1* and *GMP-PDEα2*), only one cyclic nucleotide gated ion channel (*CNG –* as opposed to two in *M. leidyi*), and the top hit to Recoverin is a *Neurocalcin-like* gene (Table 1). Neurocalcin is in the same gene family as recoverin and is also expressed in the retina but not in the rods and cones [56]. In addition, the top hit for GRK1 was G protein-coupled receptor kinase 5-like (*GRK5-like*), the top hit for RGS9–1 was regulator of G-protein signaling 12-like (*RGS12-like*), and the top hit for GC1 guanylyl cyclase were two atrial natriuretic peptide receptor 1-like (*ANPR1-like* and *ANPR1-like2*). For the rhabdomeric components, the top hit for TRP-C was an *Ankyrin-3-like* gene (Table 1). A reason for this might be that ankyrin repeats are part of TRP channels but *H. vulgaris* is likely missing a TRP ortholog [57]. Lastly, for shared components, *H. vulgaris* differed from *M. leidyi* in that we found three Visual G beta genes (*Gβ1*, *Gβ2*, and *Gβ3*) (Table 1).

**Table 1:** BLAST results for phototransduction genes

Protein name and query accession	e-value	Hydra gene name Fig.5	reciprocal BLAST top query	Reciprocal BLAST e-value	Accession
**Ciliary components**					
G-alpha-s subunit JX564543	7.00E-125	Gαs	G protein a subunit 1, partial [Hydra vulgaris]	6.00E-178	BAA81693.1
G-alpha-i subunit JQ724654	4.00E-137	Gαo	PREDICTED: guanine nucleotide-binding protein G(o) subunit alpha-like [Hydra vulgaris]	0.0	XP_002164313.3
Transducin G-alpha-t1 JX564546	2.00E-134	Gtαi	PREDICTED: guanine nucleotide-binding protein G(i) subunit alpha [Hydra vulgaris] XP_012557495.1	0.0	XP_012557495.1
Transducin G-gamma-t1 JX564547	3.00E-03	Gtɣ1	PREDICTED: guanine nucleotide-binding protein subunit gamma-like [Hydra vulgaris]	4.00E-50	XP_012561332.1
Transducin G-gamma-t1 JX564547	1.10E-02	Gtɣ2	PREDICTED: guanine nucleotide-binding protein subunit gamma-like [Hydra vulgaris]	5.00E-48	XP_002160496.1
GRK1 G protein-coupled receptor kinase 1 JX564550	1.00E-161	GRK5-like	PREDICTED: G protein-coupled receptor kinase 5-like [Hydra vulgaris]	0.0	XP_002170698.2
GMP-PDE alpha rod JX564548	4.00E-119	GMP-PDEα1	PREDICTED: dual 3′,5′-cyclic-AMP and -GMP phosphodiesterase 11A-like [Hydra vulgaris]	0.0	XP_012556304.1
GMP-PDE alpha rod JX564548	8.00E-115	GMP-PDEα2	PREDICTED: dual 3′,5′-cyclic-AMP and -GMP phosphodiesterase 11-like [Hydra vulgaris]	0.0	XP_012559729.1
GMP-PDE beta rodP23440.3	6.00E-101	GMP-PDEβ	PREDICTED: cGMP-specific 3′,5′-cyclic phosphodiesterase-like [Hydra vulgaris]	0.0	XP_012566186.2
GMP-PDE delta JX564549	9.00E-57	GMP-PDEδ	PREDICTED: retinal rod rhodopsin-sensitive cGMP 3′,5′-cyclic phosphodiesterase subunit delta-like [Hydra vulgaris]	6.00E-85	XP_012566625.1
Phosphodiesterase JQ724657	4.00E-106	GMP-PDE9A-like	PREDICTED: high affinity cGMP-specific 3′,5′-cyclic phosphodiesterase 9A-like, partial [Hydra vulgaris]	0.0	XP_002164570.3
Cyclic nucleotide gated ion channel JX564544	6.00E-77	CNG	PREDICTED: cyclic nucleotide-gated cation channel alpha-3-like isoform X1 [Hydra vulgaris]	0.0	XP_012555740.1
Cyclic nucleotide gated ion channel JX564545	6.00E-77	CNG	PREDICTED: cyclic nucleotide-gated cation channel alpha-3-like isoform X1 [Hydra vulgaris]	0.0	XP_012555740.1
RGS9–1 regulator of G-protein signaling 9 isoform 1 JX564552	8.00E-23	RGS12-like	PREDICTED: regulator of G-protein signaling 12-like [Hydra vulgaris]	0.0	XP_012555234.1
GC1 guanylyl cyclase GC-E precursor JX564553	0.0	ANPR1-like	PREDICTED: atrial natriuretic peptide receptor 1-like isoform X1 [Hydra vulgaris]	0.0	XP_004209910.2
GC1 guanylyl cyclase GC-E precursor JX564553	5.00E-156	ANPR1-like2	PREDICTED: atrial natriuretic peptide receptor 1-like [Hydra vulgaris]	0.0	XP_012560931.1
Recoverin JX564551	2.00E-121	Neurocalcin-like	PREDICTED: neurocalcin homolog [Hydra vulgaris]	1.00E-137	XP_002159500.2
**Rhabdomeric components**					
G-alpha-q subunit JQ724653	6.00E-128	Gαq	PREDICTED: guanine nucleotide-binding protein G(q) subunit alpha [Hydra vulgaris]	0.0	XP_012554580.1
Phospholipase C JQ724649	0.0	PLC	PREDICTED: 1-phosphatidylinositol 4,5-bisphosphate phosphodiesterase classes I and II-like [Hydra vulgaris]	0.0	XP_012559691.1
Trp-C protein JQ724656	7.00E-17	Ankyrin-3-like	PREDICTED: ankyrin-3-like, partial [Hydra vulgaris]	0.0	XP_004208115.1
**Shared components**					
Visual G beta JQ724652	0.0	Gβ1	PREDICTED: guanine nucleotide-binding protein G(I)/G(S)/G(T) subunit beta-1 [Hydra vulgaris]	0.0	XP_004209643.2
Visual G beta JQ724652	0.0	Gβ2	PREDICTED: guanine nucleotide-binding protein G(I)/G(S)/G(T) subunit beta-1-like [Hydra vulgaris]	0.0	XP_002164667.1
Visual G beta JQ724652	5.00E-135	Gβ3	PREDICTED: guanine nucleotide-binding protein G(I)/G(S)/G(T) subunit beta-1-like [Hydra vulgaris]	0.0	XP_002158484.1
Rhodopsin kinase JQ724650	2.00E-92	Rh kinase	PREDICTED: beta-adrenergic receptor kinase 2-like [Hydra vulgaris]	0.0	XP_012559801.1
Arrestin JQ724651	5.00E-98	Arrestin	PREDICTED: beta-arrestin-1-like [Hydra vulgaris]	0.0	XP_002158192.2
Retinal-binding protein JQ724655	9.00E-100	SEC14-like	PREDICTED: SEC14-like protein 5 [Hydra vulgaris]	0.0	XP_012563299.1

We next looked at the expression patterns of phototransduction genes to see whether they have similar expressions to the opsins. We identified a group of genes that contained most of the necessary components of the phototransduction cascade and two opsins (Additional file 3: Figure. S3). This finding provides candidate genes that function together in transducing a signal. This group had genes with high expression in the tentacle and hypostome and increasing expression during budding. This group included *ANPR1 − like2*, *GMP − PDEα2*, *Gαq*, *Gtαi*, *Gβ1*, *SEC14 − like*, *Arrestin*, *Neurocalcin − like*, *Gαs*, *GRK5 − like*, *GMP − PDEδ*, *PLC*, *GMP − PDEα1*, *ANPR1 − like*, and *Rh kinase* (Fig. 5b). When visualized together with the opsins, two opsins *HvOpC5* and *HvOpD1* had similar expression patterns to these genes (Additional file 3: Figure S3). If similar expression patterns in these genes means that they are expressed together, then these results imply that *H. vulgaris* is using components from both ciliary and rhabdomeric receptors to transduce a signal (see Discussion).

### Differentiation trajectories clustering

**Fig. 6:**
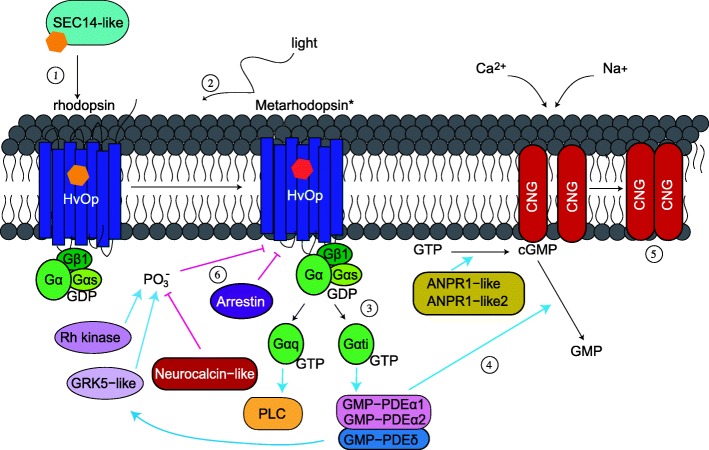
Proposed model of *H. vulgaris* phototransduction cascade. 1) SEC14-like transports a chromophore molecule (represented by a hexagon) to the opsin protein. 2) Light causes a conformational change in the chromophore structure converting rhodopsin to activated metarhodopsin. 3) Gtαi and Gαq activate GMP − PDEα1 or GMP − PDEα2 and PLC, respectively. 4) GMP − PDEα1 and/or GMP − PDEα2 convert cGMP to GMP while ANPR1-like and ANPR1-like2 convert GTP to cGMP. 5) Decrease of cGMP cause the CNG ion channel to close. 6) Metarhodopsin is deactivated by arrestin or phosphorylation by Rh kinase and GRK5-like. GMP − PDEδ has a positive interaction with GKR5-like but neurocalcin-like inhibits phosphorylation and deactivation of metarhodopsin. All genes in this figure are expressed in similar patterns. Blue arrows denote positive interactions or activation while pink lines with flat endings represent inhibition

To further detect in which cell types phototransduction genes are likely expressed, we determined to which gene clusters they belong in a stem cell differentiation trajectories clustering by Siebert et al. [52]. We expected to see phototransduction genes and one or more opsins expressed in similar cell clusters. We did not find unique matches for all of our opsin genes, but we were able to determine in which clusters 19 of them are expressed (Table 3). We only listed the top clusters, which we selected based on higher expression and expression in more cells in a cluster. We found that most opsins clustered as cells of the neuronal cells of the interstitial lineage in the endoderm or ectoderm (Table 3). *HvOpA1* and *HvOpB1* again were expressed in many more cells and cell clusters. *HvOpA1* had the densest expression in clusters of the nematocyte and nematoblast of the interstitial cell lineage (Table 3). *HvOpB1* was expressed more heavily in granular mucous gland cells and spumous mucous gland cells of the interstitial lineage (Table 3). Unlike the opsins, most phototransduction genes were expressed in all cell clusters (Additional file 4: Table S3). Two of the genes that were not expressed in all clusters were *CNG* and *GMP-PDEα1* which were expressed in neuronal ectoderm and endoderm cells of the interstitial cell lineage similar to *HvOpC5* and *HvOpD1* which we predict might be functioning together in phototransduction (Fig. 6; Table 3;Additional file 4: Table S3).

## Discussion

We present a study in which we characterize the number, location, and expression of opsin genes in the *H. vulgaris* body map, during regeneration and during budding. By using an improved genome assembly, we found that many *H. vulgaris* opsins are located near each other implying some evolution by tandem duplications. In addition, improved gene models and corrected sequences allowed us to generate a new cnidarian opsin phylogeny that supported previous claims that these genes are evolving by lineage-specific duplications. RNA-seq data for different adult tissues and time points during budding and regeneration revealed absolute and relative expression patterns for all opsins for the first time. Furthermore, by incorporating phototransduction-related genes in our opsin study, we were able to determine which genes might be involved in transducing a signal of activated opsins.

### *H. vulgaris* opsin evolution by tandem duplication

The rise of new genes, gene duplications and rapid gene expansions are often driven by tandem duplications, retrotranspositions, or chromosomal to whole genome duplications [58, 59]. Many of the genes that arise through these mechanisms acquire mutations and become pseudogenes, which are silenced, deleted, or occasionally acquire a new function [59]. Some gene copies each share the responsibility of the original gene via subfunctionalization or the new copy can perform a new function due to neofunctionalization [59]. Opsin phylogenies often group genes based on opsin type (rhabdomeric R-opsin, ciliary C-opsin, retinal G protein-coupled receptor RGR, etc.) or predicted wavelength detected (short-, medium-, and long-wavelength) [1, 3]. In the case of cnidarians, previous studies suggested that opsins are evolving by lineage-specific duplications because opsins group together by species [28, 29]. By generating a phylogenetic tree with curated *H. vulgaris* sequences, we found support for this claim. We found that opsins in the cnidarians that we included formed groups by species.

As we have access to an improved genome assembly and expanded gene models, we were able to investigate where the opsin genes are located on scaffolds. In this way, we discovered that many are near each other on the genome as well as branch near each other on the phylogeny, which implies similarity in sequences. These findings suggest that some opsin genes in *H. vulgaris* are evolving by tandem duplications. Moreover, as the assembly we used is improved but still lacks a chromosome-level contiguity, it is possible that more of the genes are close together if the scaffolds map near each other on chromosomes. It is not surprising that we see variation in the number of opsins between cnidarian species because opsins are among the phototransduction genes that have many gains and losses between clades [60]. In ray-finned fish, tandem duplications are the main mechanism by which opsin genes have evolved [61]. It would be interesting to investigate whether opsins in the other cnidarians are evolving by similar mechanisms. Specifically, in *Nematostella* and *Tripedalia* (which have a reference genome assembly) opsin gene accession consecutive numbers group next to or near each other. If these opsin genes in other cnidarians are located in close proximity it would provide support that opsin genes are rapidly evolving by lineage-specific tandem duplications in Cnidaria.

### Ctenophore or Placozoa as root of tree

One of the major discussions in determining opsin gene evolution is what to use as an outgroup or where to root the tree. It was recently discovered that ctenophores, which are more basal than cnidarians (Fig. 1b), possess opsins [27]. Prior to these findings, it was believed that cnidarians were the most basal group to have opsin-based phototransduction [24]. Although these new findings would make ctenophore opsins a good choice for outgroup, Feuda et al. argue that Placozoa opsins (placopsins) make a better outgroup [54]. Placopsins were found to be sister to all other animal opsins [3]. We generated trees using ctenophore opsin *Mnemiopsis opsin3* and Placozoa opsins as the root of the tree. We found that the tree using Placozoa opsins as root was a better option in visualizing the *H. vulgaris* opsins. By using placopsins as the root, we were able to detect the grouping of ciliary, rhabdomeric and Go/RGR opsins, and the grouping of cnidarian opsins that had been previously described [28, 29, 54].

### Location and expression of HmOps2

*H. magnipapillata opsin 2* (*HmOps2*) here referred to as *HvOpD5*, based on sequence in ﻿Plachetzki et al. [26], has been shown to be of great importance because it mediates cnidocyte discharge in *Hydra* [25]. Cnidocytes are complex cells specific to cnidarians that expel a cnidae which entwines, pierces or sticks to a target [62]. *Hydra* uses cnidocytes to immobilize prey, for movement, and as a defense against predation [62]. *Hydra* tentacles have structures called battery complexes that contain cnidocytes and sensory neurons together [25]. In situ hybridization studies found that *HmOps2*, *CNG*, and *Arrestin* co-localize in these battery complexes [25]. In addition, light was found to have an effect on how many cnidocytes are fired [25]. These results suggest that cnidocytes are firing by opsin-based phototransduction [25]. Based on these results, we expected *HvOpD5* and other phototransduction cascade genes to be upregulated in the tentacles. *HvOpD5* is located in group D in our phylogeny which is the most numerous group of *H. vulgaris* opsins. However, in terms of TPM, we did not find expression for this gene in any of our samples. It is possible that this gene is expressed at very low levels and that is why we do not detect its expression even if there is in situ evidence of its existence, location and function. Moreover, in the single cell clusters this gene is found in ectodermal neuronal cells and no expression is detected in nematocytes or nematoblast cells as would be expected.

### *HvOpA1* and *HvOpB1*

In this paper, we discovered two *H. vulgaris* opsin genes that group outside of what is expected. We had expected all opsins to form two groups as was found in previous studies [28, 29]. The reason that the grouping of these genes was not previously described is because these two genes were not detected in previous studies. The grouping is unexpected not only because we anticipated tandem evolution to be reflected by gene expansions, but also because these two genes group outside of the Hydrozoa opsins. The two genes that we found outside of the predicted clustering (groups C and D) were named *HvOpA1* and *HvOpB1*. *HvOpA1* groups together with placozoan opsins and is the most highly expressed opsin gene in *H. vulgaris* (Fig. 1; Fig. 2). *HvOpA1* is expressed everywhere but more highly in the body column and budding zone relative to other tissues (Fig. 4;Additional file 1: Fig. S1). The high expression of this gene implies that it carries an important function and thus is being actively transcribed throughout the *H. vulgaris* body.

In terms of phototransduction cascade genes, we found that *Gβ2*, *Gβ3*, and *GMP-PDE9A-like* are expressed in similar patterns to *HvOpA1* (Additional file 3: Figure. S3). One of the differences between *H. vulgaris* and *M. leidyi* was that *H. vulgaris* had 3 visual G beta genes rather than 1. Due to their expression, it is possible that *HvOpA1* functions as a G protein coupled receptor through the actions of *Gβ2* and/or *Gβ3* and the signal is continued by *GMP-PDE9A-like*. However, since *HvOpA1* is highly expressed throughout the *H. vulgaris* body and lacks the retinal-binding lysine, we do not expect it to function in light-detection. Placozoa opsins, which this gene has a similar sequence to, have yet to be characterized but are believed not to function in light detection because they also lack a retinal-binding lysine [3]. A study of opsins lacking the retinal-biding lysine in ﻿*Halobacteriacea* found that many of these genes may be non-retinal sensory opsins and some may function in chemotaxis or stress response [63]. As mentioned in the introduction, opsins can function in detecting other sensory inputs and the downstream transduction of non-visual opsins remains speculative [9]. Stem cell differentiation trajectories clustering suggests that this gene might have a function in the nematoblast and nematocyte. It is possible that *HvOpA1* functions in sensory detection at the nematocyte through a signal transduction that makes use of phototransduction cascade-like components.

Similarly, *HvOpB1* is found outside of the two main opsin groups and is one of the more highly expressed opsin genes (Fig. 1; Fig. 3). *HvOpB1* is located outside of the Hydrozoan opsins but within what could be considered the cnidopsins [29]. As a sister to other Hydrozoan opsins and its placement next to *Mnemiopsis opsin3* suggests that *HvOpB1* may be a more ancestrally derived gene and that its function may be conserved in orthologous genes in other species. *HvOpB1* is upregulated in the hypostome and increases in expression during budding. This implies that this gene has a potential role in the *H. vulgaris* head (Fig. 3; Additional file 1: Figure S1). Again, *HvOpB1* also lacks the conserved retinal-binding lysine (Table S1). Stem cell differentiation trajectories clustering suggests that this gene might have a function in mucous gland cells. As we know that opsins can take on roles in other types of sensory perception such as heat and sound [9], it is possible that *HvOpB1* may function in detecting something other than light. If *HvOpB1* functions in sensory perception it may be used to detect prey, or it may function in digestive enzyme secretion near the *H. vulgaris* mouth.

As mentioned above, *HvOpA1* and *HvOpB1* group outside of the Hydrozoan opsins and lack the conserved retinal-binding lysine. These results show that *HvOpA1* and *HvOpB1* are single copies. A simple model for *H. vulgaris* opsin evolution would show that there are 4 opsin genes, two giving rise to group C and D by gene duplications. An implication is that opsins with a retinal-binding site are more susceptible to gene expansions. This is the case if a new opsin gene arises and is maintained by subfunctionalization or neofunctionalization. Retention of these genes is not surprising because animals with color vision require multiple opsin genes with overlapping wavelengths, which are determined by variation in opsin sequence. The maintenance of *HvOpA1* and *HvOpB1* as single genes implies that they have a function which requires a precise sequence and would not benefit from the rise of a new gene. In addition, the placement of these genes, *HvOpA1* with Trichoplax opsins and *HvOpB1* near ﻿*Mnemiopsis* opsins, suggests they may be ancestral. These genes might serve a sensory function that preceded the role of opsins in vision. Future studies should investigate the function of *HvOpA1* and *HvOpB1* through *in situ*s or knockout experiments.

### Potential phototransduction cascade in *Hydra*

When looking for phototransduction cascade genes in *H. vulgaris*, we noticed that one of the differences between this species and *M. leidyi* was that some of the genes had more copies in *H. vulgaris*. Genes that have two or more copies might have related function in transduction via subfunctionalization or may have new functions due to neofunctionalization [58]. We expected to find ciliary components because cnidarian opsins are similar to vertebrate ciliary opsins, yet we found homologs of both rhabdomeric and ciliary components (Table 1). The only gene that we did not find a homolog to was TRP and instead we found only one copy of CNG, which is the ion channel that cnidarian opsins should function by [24].

We hypothesized that genes functioning together in a cascade would be co-expressed. Our hypothesis was correct as many of the phototransduction-like genes that we identified grouped together as having similar expression patterns (Fig. 5b). These genes have similar expression patterns to *HvOpC5* and *HvOpD1* which are more highly expressed in the hypostome and tentacle relative to other tissues and increase in expression at later stages of budding and regeneration (Additional file 1: Figure S1; Additional file 2: Figure S2). Since these opsin genes are expressed at similar patterns to other phototransduction cascade-like genes, it is possible that these opsins function in light detection. Under the assumption that similar expression patterns imply that these genes work together, we have come up with a potential phototransduction cascade in *H. vulgaris* (Fig. 6). *H. vulgaris* SEC14-like transports the chromophore to bind opsins (*HvOpC5* and/or *HvOpD1*) and become inactive rhodopsin to be activated by light. Activation of rhodopsin to metarhodopsin proceeds by actions of Gαq or Gtαi that forms a complex with Gαs and Gβ1. Gtαi binds GMP − PDEα1 or GMP − PDEα2 to transduce the signal while Gαq activates PLC. Although CNG does not group with these genes in the heatmap and has different patterns of expression, it is likely the channel causing the cell to hyperpolarize. Cyclic GMP (cGMP) is bound to CNG maintaining the channel open. GMP − PDEα1 and/or GMP − PDEα2 convert cGMP to GMP which closes the CNG ion channel. Conversely, ANPR1-like and ANPR1-like2 [64, 65] convert GTP to cGMP helping to regulate the opening and closing of CNG. Finally, transduction is terminated by arrestin deactivating metarhodopsin and phosphorylation of metarhodopsin by Rh kinase and GRK5-like. Deactivation of metarhodopsin is also regulated by neurocalcin-like which inhibits phosphorylation of light-activated rhodopsin [66] and GMP − PDEδ which activates GKR5-like [67]. It is also important to note that our hypothetical *H. vulgaris* phototransduction cascade includes aspects of both rhabdomeric and ciliary receptors. We base this model off of evidence that these genes are being expressed in similar tissues and patterns. It is possible that the opsin transduction cascade in basal lineages use components that were later specialized with some loss in rhabdomeric and ciliary opsins through co-option [68]. Some support for this comes from ﻿*Mnemiopsis leidyi* having RNA evidence for rhabdomeric and ciliary phototransduction components and *Nematostella* opsin group 1 branching together with rhabdomeric opsins while we would expect cnidarian opsins to be closer to ciliary.

## Conclusions

We provide the first study to characterize opsin genes in *H. vulgaris* using RNA-seq data for different tissues and time points during regeneration and budding. Previous studies have focused on reconstruction of the opsin gene phylogeny but did not explore the expression patterns of these genes. Gene expression can give us an insight into the potential function of some of these genes that are rapidly evolving. While our phylogenetic tree was very similar to those of previous studies, we discovered two *H. vulgaris* opsins that were outside of the typical two groups of opsins. One of these genes was the most highly expressed opsin in *H. vulgaris* and the other was highly expressed in the hypostome and increased in expression during budding. By using the improved genome assembly (v2.0) and improved gene models, we found that opsin genes in *H. vulgaris* are likely evolving by tandem duplications. These results can be combined with opsin gene mapping in other cnidarians to see if opsin genes might be evolving by the same mechanism in the entire phylum which would explain the linage-specific duplications. Furthermore, by combining opsin expression data with that of phototransduction-related genes, we were able to generate a model for phototransduction in *H. vulgaris*. Future work will focus on the morphological and behavioral effects of turning off some of these candidate genes.

Our results are of interest to the fields of genome evolution, cnidarian biology and evolution of vision. For one, we find a sensory gene family that is likely evolving by tandem duplications. The evolution of opsin genes has been of particular interest and we provide a suggested mechanism for gene expansions. In cnidarian biology, the function of the opsin genes is only known for *HmOps2* (*HvOpD5*), which is involved in cnidocyte firing. Yet, although *Hydra* lack eyes, they respond to light so it is possible that one or more of the opsins function in phototransduction. Opsins have also been shown to have functions in other sensory detection such as heat or sound and the number of opsins in *Hydra* make it a possibility that some of the genes may have other sensory functions. Thus our general characterization of molecular evolution and gene expression should serve as a foundation for future studies of non-ocular cnidarian opsin gene functions.

## Methods

### RNA extractions and library preparation

*Hydra vulgaris* polyps were kept in *Hydra* medium (1 M CaCl_2_, 0.1 M MgCl_2_, 0.03 M KNO_3_, 0.5 M NaHCO_3_, 0.8 M MgSO_4_) and were fed freshly hatched *Artemia salina* nauplii twice per week. Before RNA isolation, animals were starved for at least 1 day. For the head regeneration time course, 1 animal per sample was bisected and allowed to undergo head regeneration for 0, 2, 4, 6, 12, 24, or 48 h. The apical region of the animal was once again bisected, and the tissue was used for RNA extraction. For the budding experiment, the head region of buds at stages S1, S3, S4, S5, S6, S7, S8, or S10 of budding was bisected and used for total RNA extraction. Adult polyps were dissected to separate the tentacles, budding zone, body column, hypostome, and foot for RNA extraction.

Tissues were dissolved in RLT buffer (Qiagen RNeasy) with 2- betamercaptoethanol within 3 min of isolation. RNA was extracted using Qiagen RNeasy kit according to the manufacturer’s protocol and treated with DNase from TURBO DNA-free kit. Agilent Bioanalyzer was used to check RNA quality. Only samples with RIN scores ≥9 were used for RNA-seq library preparation.

A modified Smart-seq2 protocol was used to build RNA-Seq libraries (Picelli et al., 2014). Poly-dT primer and reverse transcriptase were used to generate full-legth cDNA from mRNA. The number of PCR cycles used to amplify cDNA was based on the initial amount of total RNA. 20 ng of cDNA was used to make sequencing library using a tagmentation enzyme (Illumina Nextera kit) amplified for 8 cycles of PCR. Libraries were multiplexed and sequenced as 43 bp Illumina paired-end reads GEO accession GSE127279 [42].

### Identification of *Hydra* opsin genes

An ab initio reference transcriptome for *Hydra vulgaris* was assembled using RNA-seq libraries built as described above. To generate an ab initio transcriptome, adapter sequences and low quality base pairs from the paired-ends reads were trimmed using Trimmomatic v. 0.35 [69] with the following parameters: “PE [read1.fastq] [read2.fastq] pe_read1.fastq.gz se_read1.fastq.gz pe_read2.fastq.gz se_read2.fastq.gz ILLUMINACLIP:NexteraPE-PE.fa:2:30:8:4:true LEADING:20 TRAILING:20 SLIDINGWINDOW:4:17 MINLEN:30”. The trimmed reads were mapped to the *Hydra* 2.0 genome using STAR v. 2.4.2a [70] with the following parameters: “--outFilterMultimapNmax 20 --alignSJoverhangMin 8 --alignSJDBoverhangMin 1 --outFilterMismatchNmax 999 --outFilterMismatchNoverReadLmax 0.04 --alignIntronMin 20 --alignIntronMax 1000000 --alignMatesGapMax 1000000 --outSAMunmapped Within --outFilterType BySJout --outSAMattributes NH HI AS NM MD XS --outSAMstrandField intronMotif --outSAMtype BAM SortedByCoordinate --sjdbScore 1”. Mapped reads from two biological replicates for each sample were pooled and ab initio transcripts were assembled using StringTie v. 1.3.4b [71] with the parameters: “-G [GTF file] -o stringtie.gtf -c 3 -p 12 –A stringtie.abundance.txt”. Assembled transcripts for all samples were then merged with the *Hydra* 2.0 gene models to obtain a final reference transcriptome (Additional file 5: Table S4). The reference transcriptome was annotated using Blast2GO [72]. BLAST search was done for the transcripts against NCBI’s non-redundant NR database. The transcripts were then annotated with the gene ontology (GO) terms associated with the BLAST hits using the “Mapping” and “Annotation” of Blast2GO. The GO terms were further expanded using InterProScan and Annex mapping utilities of Blast2GO.

We searched the gene annotations for GO terms relating to opsins and extracted the sequences from those genes. In addition, we extracted *Hydra* opsin sequences from the phylogenetically-informed annotation (PIA) database [53]. All sequences were aligned to the *H. vulgaris* reference genome 2.0 [11] with command-line BLAST using blastn -query opsin_seqs.txt -db hydra_stringtie_merged -evalue 1e-10 -outfmt 6 [73] for a full list of candidate opsin genes. Blast output allowed us to manually correct incomplete transcriptome sequences possibly due to misalignments. Manually corrected sequences were aligned back to the genome for verification. Sequences were visually inspected and a neighbor joining tree was generated in MEGA7 using a Poisson model of substitution and gamma distributed rates among sites [74] to identify and retain unique *Hydra* opsin sequences. Opsin genes were named based on phylogenetic grouping based on (Fig. 2) then location on scaffolds (Additional file 4: Table S1).

### Opsin phylogeny

In addition to the *H. vulgaris* genes determined above, we extracted opsin sequences for cnidarians *Podocoryna carnea*, *Cladonema radiatum*, *Tripedelia cystophora*, and *Nematostella vectensis* from NCBI GenBank. For a more complete phylogenetic tree, we included opsin sequences for ctenophore *Mnemiopsis leidyi* from [27] and sequences for *Trichoplax adhaerens*, *Drosophila melanogaster* and *Homo sapiens* from [54]. All opsin sequences were aligned in MEGA7 using MUSCLE [75]. The alignment was visually inspected and manually adjusted. We determined the best fit our data by calculating Bayesian Information Criterion (BIC) [74, 76]. To do this, we used the model selection tool in MEGA7 as follows: automatic (neighbor-joining tree) as a tree to use, maximum likelihood statistical method, and amino acid substitutions type using all sites. The best model was LG + G + F with a BIC value of 129,956. We then ran the phylogeny reconstruction in MEGA7 as follows: maximum likelihood statistical method, bootstrap method as a test of phylogeny with 100 replicates, amino acid substitution type, model LG with Freqs. (+F), gamma distributed (G) rates among sites with 5 discrete gamma categories, using all sites, and Nearest-Neighbor-Interchange (NNI) as the heuristic method.

### Identification of phototransduction genes

Sequences for phototransduction genes in *Mnemiopsis leidyi* were obtains from NCBI GenBank according to accession numbers in Table 2 of ﻿Schnitzler et al. [27]. These sequences were aligned against our *H. vulgaris* StringTie assembly using command-line BLAST [73]. Sequences were extracted and BLAST against NCBI GenBank using Blastx with default parameters to obtain a top hit. Expression data for these genes was extracted from a TPM counts matrix (generated as described in *Identification of* Hydra *opsin genes*) and visualized using heatmap3 [77].

**Table 2:** Differentiation trajectories clustering^†^

Opsin	Juliano aepLRv2 ID	Cluster
HvOpA1	t29274aep	i_nb2, i_nb3, i_nb4, i_nb5, ecEp_bd, i_nem
HvOpB1	t21413aep	i_smgc1, i_smgc2, i_gmgc, i_n_en2
HvOpC1	t26793aep	i_n_ec1, i_n_ec3, i_n_ec4
HvOpC2	t24044aep	i_n_ec1, ecEp_nem
HvOpC3	t10575aep	i_n_ec1, i_n_ec3, i_n_ec5
HvOpC4	no match*	
HvOpC5	t24564aep	i_n_ec1, i_n_ec4
HvOpD1	t36346aep	i_n_ec1, i_n_ec3, i_n_en1, i_n_en2
HvOpD2	no match	
HvOpD3	no match	
HvOpD4	t37969aep	i_n_ec1, i_n_ec3, i_en1, i_n_en2
HvOpD5	t29512aep	i_n_ec1, i_n_ec3, i_nc_prog
HvOpD6	no match	
HvOpD7	no match	
HvOpD8	t36136aep	i_n_ec1, i_n_ec3
HvOpD9	no match	
HvOpD10	no match	
HvOpD11	no match	
HvOpD12	no match	
HvOpD13	no match	
HvOpD14	t33805aep	i_n_ec1, i_n_ec3, i_n_ec4
HvOpD15	no match	
HvOpD16	no match	
HvOpD17	t27882aep	i_gmgc, i_n_ec3, i_n_en3, i_nc_gc_prog, i_smgc1, i_smgc2, i_zmg1, i_zmg3
HvOpD18	no match	
HvOpD19	no match	
HvOpD20	no match	
HvOpD21	no match	
HvOpD22	no match	
HvOpD23	no match	
HvOpD24	t32881aep	i_fmgl1, i_smgc3
HvOpD25	t36280aep	i_n_ec3
HvOpD26	t25412aep	i_n_ec1, i_n_ec3, i_n_ec4, i_n_ec5, i_n_en1
HvOpD27	no match	
HvOpD28	no match	
HvOpD29	t2106aep	i_n_ec1, i_n_ec3, i_n_en2
HvOpD30	t20043aep	i_n_ec2
HvOpD31	no match	
HvOpD32	no match	
HvOpD33	no match	
HvOpD34	no match	
HvOpD35	t29959aep	ecEp_nb, ecEp_nem, ecEp_SC1, ecEp_SC2, ecEp_bd, enEp_foot
HvOpD36	no match	
HvOpD37	no match	
HvOpD38	t27688aep	i_nem

^†^Clustering according to data from Siebert et al. [52] derived using the interactive tool at https://portals.broadinstitute.org/single_cell/study/SCP260/stem-cell-differentiation-trajectories-in-hydra-resolved-at-single-cell-resolution

*no match means no unique match to the Seibert et al. transcriptome. The top BLASt hit to these genes was a better match to another HvOp gene

Cluster Label Abbreviation Key: bat: battery cell, bd: basal disk, db: doublet cluster, ec: ectoderm, ecEP: ectodermal epithelial cell, en: endoderm, enEP: endodermal epithelial cell, fmgl: female germ-line, gc: gland cell, gmgc: granular mucous gland cell, i: cell of the interstitial lineage, id: integration doublet, mgl: male germline, mp: multiplet, nb: nematoblast, n: neuronal cell, nem: nematocyte, pd.: suspected phagocytosis doublet, prog: progenitor, SC: stem cell, smgc: spumous mucous gland cell, tent: tentacle, zmg: zymogen gland cell

### Opsin expression

RNA-seq libraries from adult *H. vulgaris* tentacles, hypostome, body column, budding zone and also from a budding and head regeneration time course at stages S1, S3, S4, S5, S6, S7, S8, S10, and at hours 0, 2, 4, 6, 12, 24 and 48 were used, GEO accession GSE127279 [42]. Adapter sequences and low quality base pairs from the paired-ends reads were trimmed using Trimmomatic v. 0.35 (see *Identification of* Hydra *opsin genes*). Trimmed reads were mapped to the reference transcriptome using bowtie v. 1.2 [78] with the following options: “-X 2000 -a -m 200 -S --seedlen 25 -n 2 -v 3” and quantified using RSEM v. 1.2.31 [79]. Batch effects were removed from gene expression levels (TPM) using “ComBat” function from sva package v. 3.18 [79] in R v. 3.2.3. TPM values were extracted for the opsin genes. TPM values were smooth quantile normalized using qsmooth package [80] in R. Normalized values were used for *HvOpA1.* Expression was visualized by generating bubble plots in ggplot2 [81] and heatmap3 [77].

### Differentiation trajectories clustering

To determine in which single cell clusters opsins and phototransduction genes were expressed, we searched the interactive browser containing data from Siebert et al. [52]. We first used BLAST in the *Hydra* 2.0 Genome Project Portal https://research.nhgri.nih.gov/hydra/sequenceserver/ to align our sequences to the Juliano aepLRv2 database. We then used these IDs to visualize the expression of these genes in different clusters at https://portals.broadinstitute.org/single_cell/study/SCP260/stem-cell-differentiation-trajectories-in-hydra-resolved-at-single-cell-resolution. Although some of the opsins are expressed in many clusters, we only listed top matches which are the clusters in which the genes had higher expression and where expressed in more of the cells in that cluster.

## Supplementary information

**Additional file 1: Figure S1.** Opsin expression in the *H. vulgaris* body, budding, and regeneration. (A) Z-scores of opsin RNA-seq expression in *H. vulgaris* compared between the body column, budding zone, foot, hypostome, and tentacles. (B) Z-scores of opsin RNA-seq expression during *H. vulgaris* budding (asexual reproduction) compared between samples from stages 1, 3, 4, 6, 7, 8, and 10. (C) Z-scores of opsin RNA-seq expression during *H. vulgaris* head regeneration comparing samples from time points 0 h, 2 h, 4 h, 6 h, 12 h, 24 h, and 48 h.

**Additional file 2: Figure S2.** Opsin expression across all samples. Heatmap showing RNA-seq z-scores across the *Hydra* body, during regeneration and during budding for the 45 opsin genes. Gene name order was ignored to allow opsins to group by expression patterns.

**Additional file 3: Figure. S3.** Heatmap of z-scores for all phototransduction genes across all samples. Heatmap showing RNA-seq z-scores across the *Hydra* body, during regeneration and during budding for the all opsins and phototransduction genes. Gene name order was ignored to allow opsins to group by expression patterns.

**Additional file 4: Table S1**. Assigned *H. vulgaris* opsin gene names and locations. **Table S2**. *H. vulgaris* opsin sequences and GenBank accession numbers. **Table S3.** Single cell clustering for phototransduction genes.

**Additional file 5: Table S4.** GTF file of combined StringTie and Hydra v2.0 gene models. (GTF 50712 kb)

## Data Availability

Opsin sequences have been deposited in GenBank under accession numbers MN822248-MN822292. Other datasets are included in the text and supplementary materials.

## Abbreviations

ANPR1-Like: atrial natriuretic peptide receptor 1-like
cGMP: Cyclic GMP
CNG: Cyclic nucleotide gated ion channel
C-Opsin: Ciliary opsin
GMP: Guanosine monophosphate
GMP-PDE: GMP phosphodiesterase
Go/RGR: Go-coupled, retinal G protein-coupled receptor
GRK: G Protein-coupled receptor kinase
Gtɣ: Transducin G-gamma
GTP: Guanosine triphosphate
Gtαi: Transducin G-alpha-i
G*α*q: G-alpha-q
Gαs: G-alpha-s
Gβ: visual G beta
HvOp: Hydra vulgaris opsin
RGS: Regulator of G protein signaling
Rh: Rhodopsin
R-opsin: Rhabdomeric opsin
TPM: Transcripts per million
TRP: Transient receptor potential channel
