# Supplementary figures and images for: Molecular evolution and expression of opsin genes in Hydra vulgaris
Source: BMC Genomics. 2019 Dec 17;20:992. doi: 10.1186/s12864-019-6349-y (PMC6918707; doi:10.1186/s12864-019-6349-y)

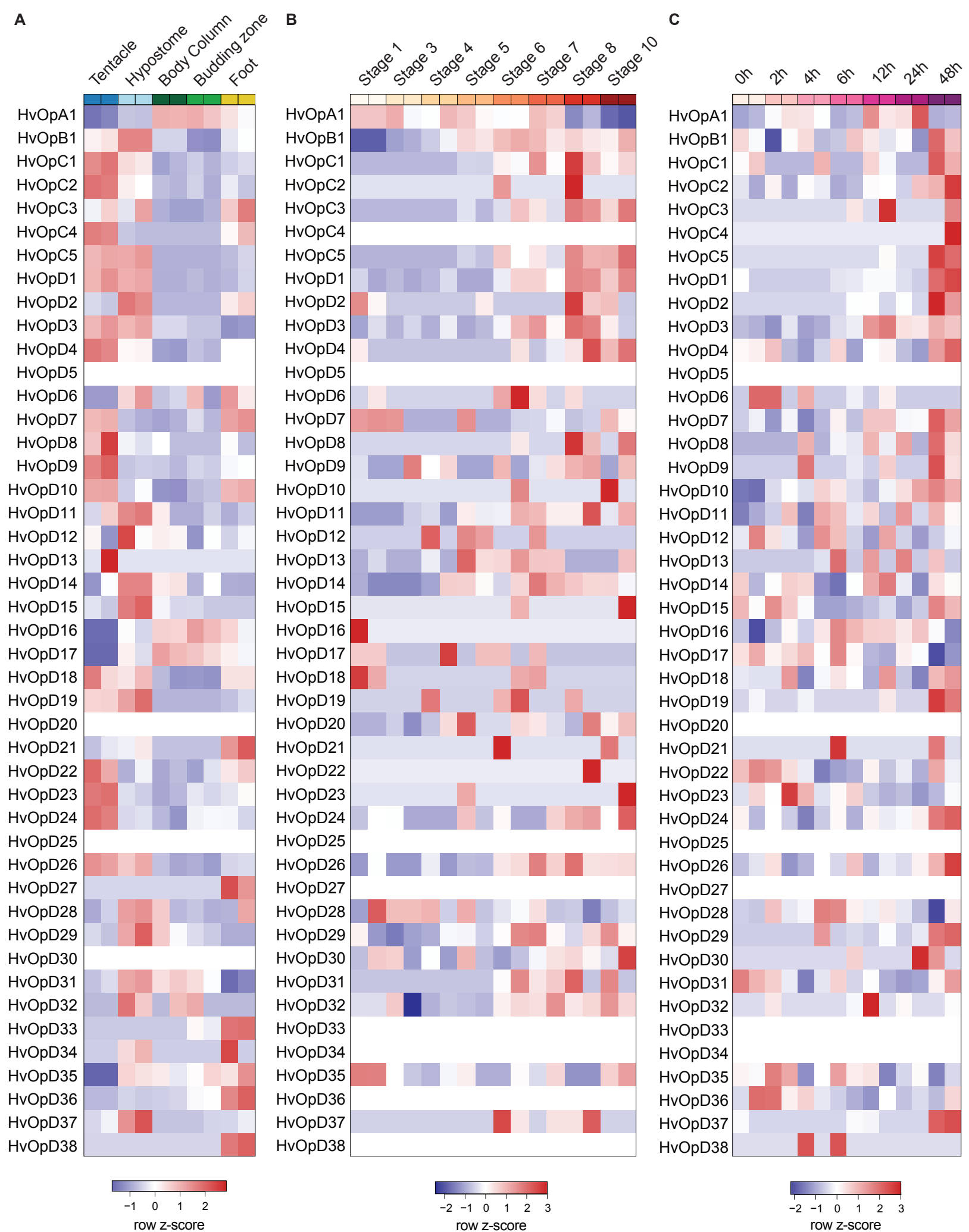

Supplement: Supplementary file 1 — Additional file 1: Figure S1. Opsin expression in the H. vulgaris body, budding, and regeneration. (A) Z-scores of opsin RNA-seq expression in H. vulgaris compared between the body column, budding zone, foot, hypostome, and tentacles. (B) Z-scores of opsin RNA-seq expression during H. vulgaris budding (asexual reproduction) compared between samples from stages 1, 3, 4, 6, 7, 8, and 10. (C) Z-scores of opsin RNA-seq expression during H. vulgaris head regeneration comparing samples from time points 0 h, 2 h, 4 h, 6 h, 12 h, 24 h, and 48 h. [file 12864_2019_6349_MOESM1_ESM.pdf]

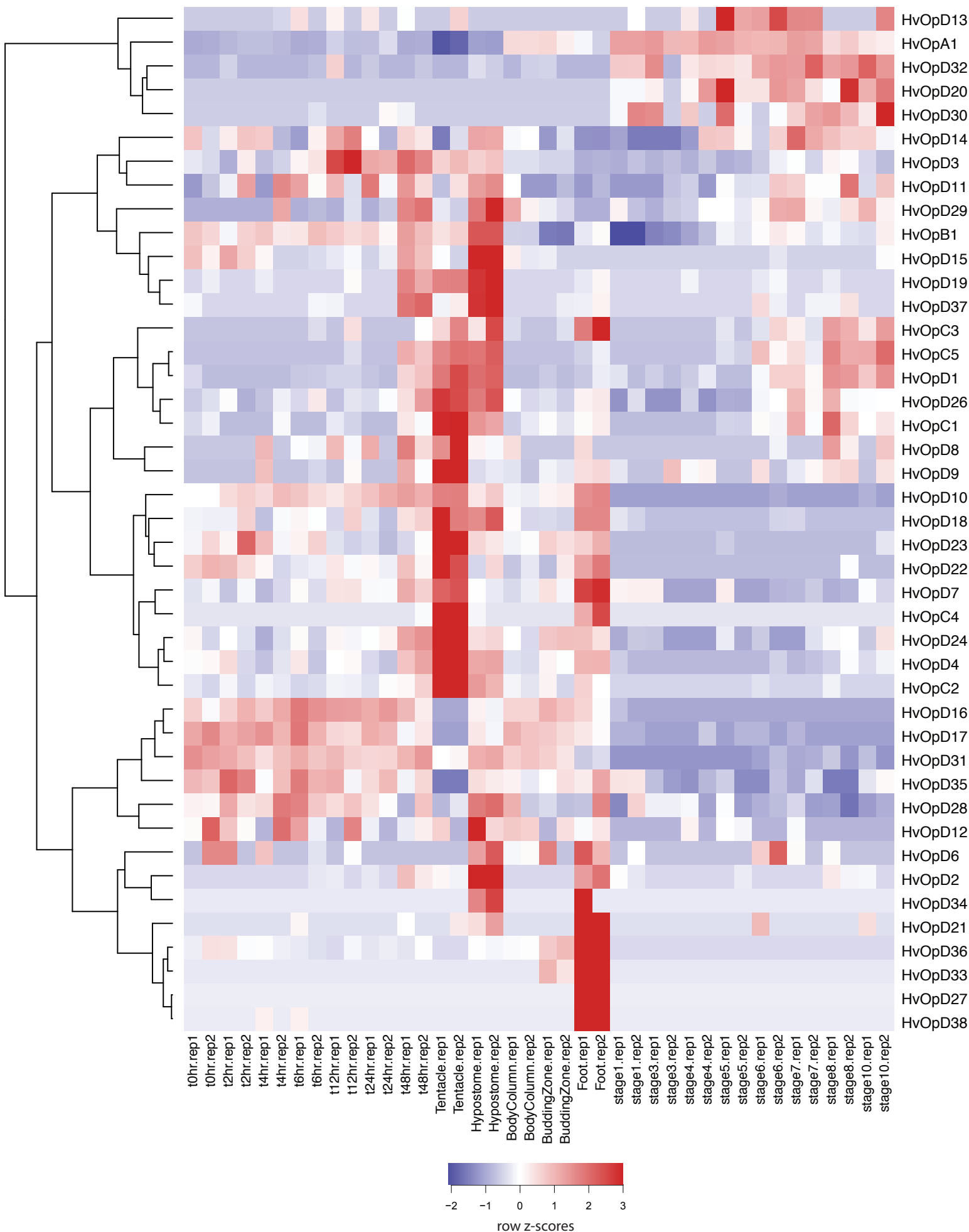

Supplement: Supplementary file 2 — Additional file 2: Figure S2. Opsin expression across all samples. Heatmap showing RNA-seq z-scores across the Hydra body, during regeneration and during budding for the 45 opsin genes. Gene name order was ignored to allow opsins to group by expression patterns. [file 12864_2019_6349_MOESM2_ESM.pdf]

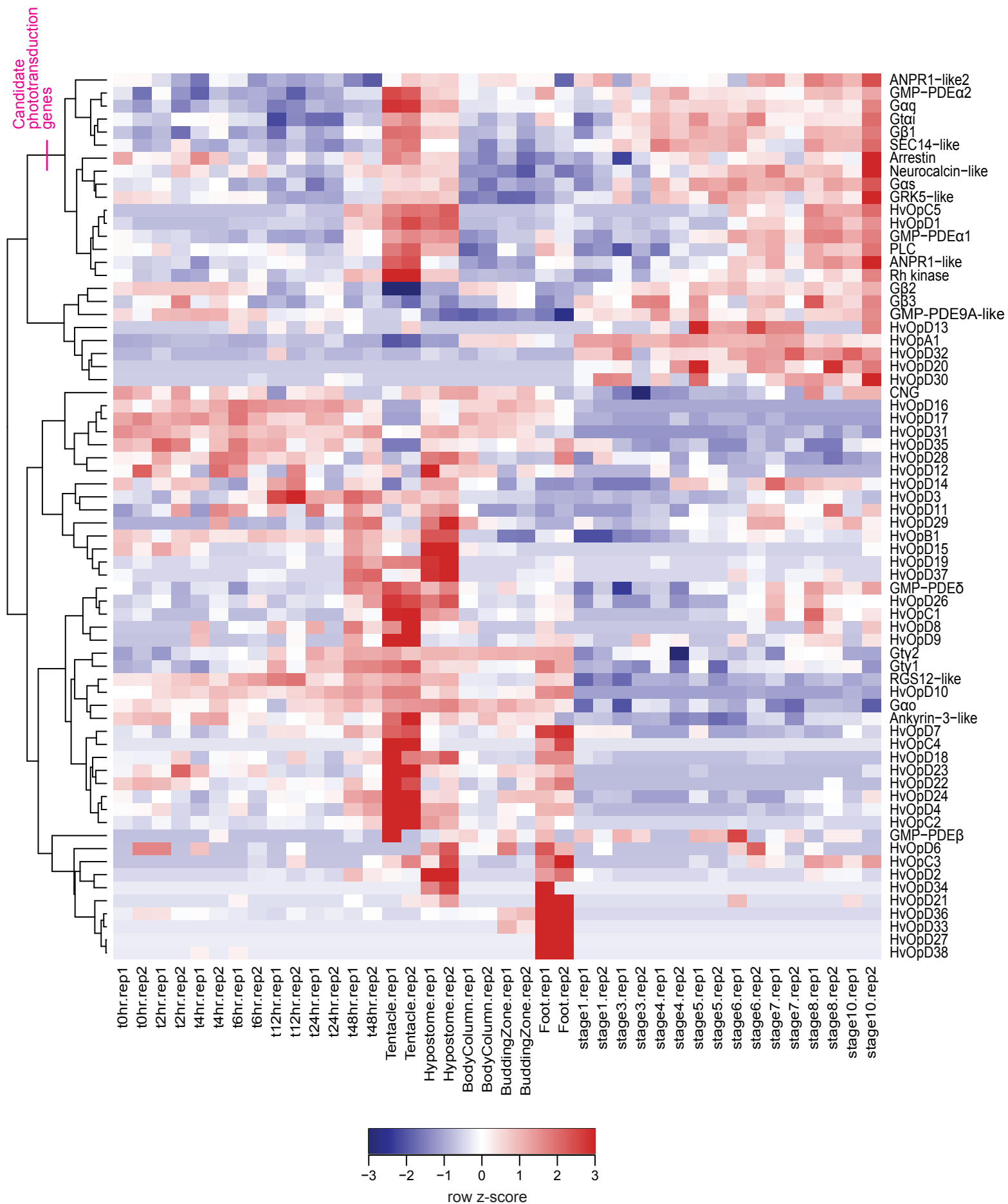

Supplement: Supplementary file 3 — Additional file 3: Figure. S3. Heatmap of z-scores for all phototransduction genes across all samples. Heatmap showing RNA-seq z-scores across the Hydra body, during regeneration and during budding for the all opsins and phototransduction genes. Gene name order was ignored to allow opsins to group by expression patterns. [file 12864_2019_6349_MOESM3_ESM.pdf]
